# A protocol of using PTMiner for quality control and localization of protein modifications identified by open or closed search of tandem mass spectra

**DOI:** 10.52601/bpr.2022.220024

**Published:** 2022-12-31

**Authors:** Zhiyuan Cheng, Ge Song, Yan Fu

**Affiliations:** 1 Academy of Mathematics and Systems Science, Chinese Academy of Sciences, Beijing 100190, China; 2 Graduate School of Peking Union Medical College, Beijing 100730, China; 3 National Institute of Biological Sciences, Beijing, Beijing 102206, China; 4 School of Mathematical Sciences, University of Chinese Academy of Sciences, Beijing 100049, China

**Keywords:** Modification identification, Open search, False discovery rate, Modification localization, False localization rate

## Abstract

In recent years, an open search of tandem mass spectra has greatly promoted the detection of post-translational modifications (PTMs) in shotgun proteomics. However, post-processing of the results from open searches remains an unsatisfactorily resolved problem, which hinders the open search mode from wide practical use. PTMiner is a software tool based on dedicated statistical algorithms for reliable filtering, localization and annotation of the modifications (mass shifts) detected by open search. Furthermore, PTMiner also supports quality control and re-localization of modifications identified by the traditional closed search. In this protocol, we describe how to use PTMiner for the two search modes. Currently, the search engines supported by PTMiner include pFind, MSFragger, MaxQuant, Comet, MS-GF + and SEQUEST.

## INTRODUCTION

### Why PTMiner?

The presence of post-translational modifications (PTMs) is a major reason for the complexity of proteome and the existence of “proteoforms”, *i*.*e*., different proteins produced from the same gene. The PTM proteoforms have the same amino acid sequences but vary in a number of specific sites that are differentially modified. PTMs on these sites may cause huge changes in the physical and chemical properties of the proteins. As a consequence, the identification of these PTMs and their precise sites is crucial to the study of protein structure and function. Mass spectrometry (MS) instruments are developing rapidly, and have greatly promoted the development of proteomics (Aebersold and Mann 2016). The PTMs are reflected as specific mass shifts of the peaks in the tandem mass spectra of peptides. Therefore, matching the experimental mass spectra with the theoretical spectra predicted from the peptide sequences (possibly in modified forms) in a protein database enables the effective identification of proteins and their PTMs. The traditional way to do this is called *Closed Database Search* which uses a tight tolerance of precursor masses and can only consider a limited number of modification types (Yates* et al.* 1995). When there exist many unanticipated or unknown modifications in the sample, the closed search may fail to interpret a large proportion of mass spectra (Fu 2016). Open search can solve this problem by using a large precursor tolerance, *e*.*g*., 500 Da. The mass shifts between the experimental spectra and the theoretical spectra are explained as potential modifications. A number of open search engines have been developed since this idea was proposed in 2000 (Pevzner* et al.* 2000). In recent years, this strategy has been greatly promoted by several newly developed search engines, *e*.*g*., Open-pFind (Chi* et al.* 2018) and MSFragger (Kong* et al.* 2017), which can analyze large-scale MS data at speeds comparable to closed search.

However, open searches produce search results that are of higher complexity and error rate than closed searches. Effective post-processing of the open-search results remains an unsatisfactorily resolved problem, which hinders the open search mode from wide practical use. Most open search engines simply report the mass shifts without localizing them to the specific amino acids on the peptides. Although some engines try to localize the mass shifts, they do this in rather simplistic ways leading to a high false localization rate. In open search, the modifications (mass shifts) can in theory occur on any site of the peptides and are thus much more difficult to precisely localize than those in closed search. The widely used target-decoy search (TDS) approach to estimate the false discovery rate (FDR) essentially evaluates the quality of peptide-spectrum matches (PSMs) at the whole peptide sequence level and cannot measure the error rate of modification sites. Moreover, even when used to control the FDR of PSMs, the TDS approach often faces some problems in PTM identification. A global FDR as estimated by current search engines is usually inapplicable to the subset of modified peptides of interest. Separate FDR estimation is a better choice but may also fail when the search result contains a very small number of PSMs with target PTMs. This problem is very common in open search which generates various mass shifts, which are mostly in small numbers. Finally, the identities of the discovered mass shifts have to be properly annotated by either matching them to existing entries in the protein modification database or classifying them as novel ones. This is a simple task but also prone to annotation mistakes.

PTMiner is a software tool we developed to address the above issues faced by the open search strategy (An* et al.* 2019). It uses dedicated statistical algorithms to effectively control the quality of PSMs with various mass shifts and confidently localize the modification sites on the peptides. It annotates the identities of mass shifts by matching them to the existing entries in the Unimod database. Tested on both simulated and synthesized-peptide datasets, PTMiner successfully controlled the FDR of peptides carrying diverse modifications using the transfer FDR method (Fu and Qian 2014). It also localized the modification sites more accurately than open search engines or existing re-localization algorithms. We used PTMiner to characterize the protein modifications (both known and unknown) in a draft map of the human proteome which contains approximately 25 million MS/MS spectra. From the open-search results from pFind (v2.8), PTMiner reported over 1.7 million modified PSMs at 1% FDR and 1% FLR (false localization rate). In addition, PTMiner can also more accurately re-localize the modifications identified by the closed search (An* et al.* 2018).

Here we list five reasons to use PTMiner. (1) Transfer FDR in PTMiner is an effective and reliable FDR estimation method for different modifications. (2) For open search, PTMiner can localize mass shifts more precisely, and can annotate the mass shifts according to the Unimod database. (3) For closed search, PTMiner can also re-localize the modifications and control their FLR. (4) Several popular search engines are supported, with seamless support of pFind3 and MSFragger. (5) PTMiner is free, fast and friendly.

### The focus of this protocol

In this protocol, we demonstrate how to post-process the modified PSMs using PTMiner. We first show the configuration and usage of PTMiner for the open search mode and then for the closed search mode. Finally, we show several parts of the result files and describe the meaning of each attribute.

### The workflow of PTMiner

**Figure 1:**
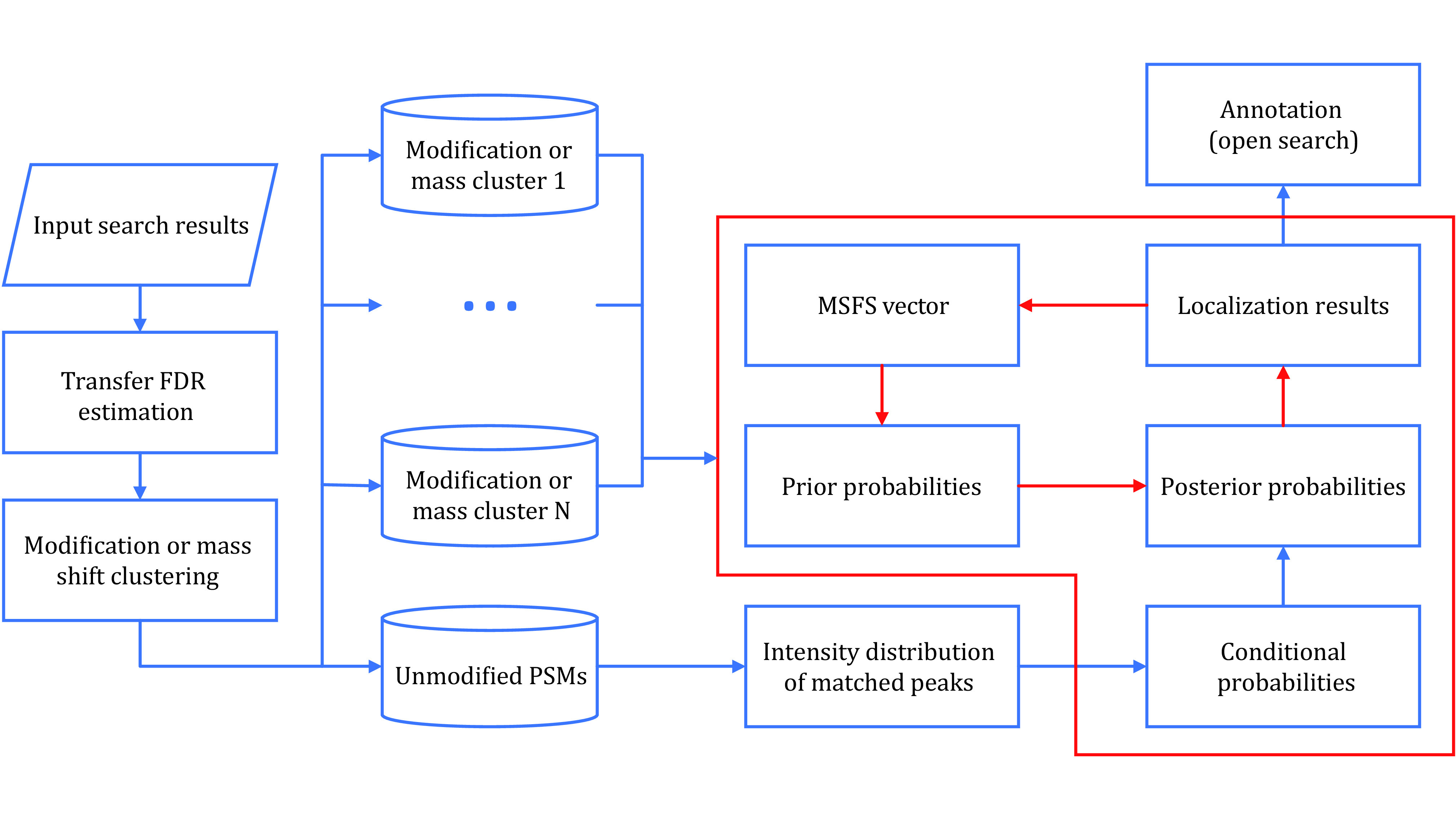
The workflow of PTMiner. The part in the red box shows the process of the localization algorithm, in which the red arrows represent iterative updating of prior probabilities

The PTMiner program contains three modules for post-processing the results of search engines. The first module is FDR filtering for both closed search and open search. The second module is mass-shift localization for open search or re-localization of modification sites for closed search. The last module is the annotation of mass shifts (only for open search). PTMiner matches the mass shifts and their specificities to the entries in Unimod. The following is the detailed process of PTMiner (see Fig. 1).

(1) For open search, PTMiner first groups PSMs by mass shifts into 1-Da-size bins centered on integers, *e*.*g*., [0.5, 1.5] Da. Then, transfer FDR (recommended) estimation is applied to each bin to yield modification-specific FDRs, and the PSMs are filtered to control the FDRs at a given level. For closed search, PTMiner groups PSMs by the target modifications specified by the user and filters PSMs using transfer FDR.

(2) For open search, PTMiner clusters the mass shifts within each bin using a Gaussian mixture model and treats each cluster as a target modification. For closed search, the target modifications are specified by the user. PTMiner then groups the PSMs (after filtration in the above step) by the target modifications. For unmodified PSMs or PSMs with non-target modifications, PTMiner groups them as one cluster to fit the distribution of matched-peak intensities for localization probability calculation.

(3) PTMiner calculates the localization probability in a Bayesian framework. It first sets uniform prior probabilities for all sites (open search) or user-specified sites (close search) and calculates a conditional probability for each candidate site on the peptide using the intensity distribution of matched peaks. From the prior probabilities and conditional probabilities, posterior probabilities are calculated, and the modification is localized to the site of the maximum posterior probability. Importantly, PTMiner uses an EM-like algorithm to iteratively update the prior probabilities to improve the localization accuracy.

(4) For open search, PTMiner compares the localized mass shifts with the modifications in the Unimod database to annotate the identities of mass shifts. The mass shifts may be fully annotated (both mass and site matched), partially annotated (only mass matched), or unannotated.

## SETUP

### Hardware and software requirements

• A personal computer (PC) with at least 2 GB of RAM

• 64-bit version of Microsoft Windows 7 or a newer operating system

• More than 740 pixels in the vertical direction of the computer display resolution.

• .NET framework 4.5 or a higher version. Available at: https://www.microsoft.com/en-us/download/details.aspx?id=30653

• PTMiner 1.2.6 or higher. Free download at http://fugroup.amss.ac.cn/software/PTMiner/PTMiner.html

### Installation of PTMiner

No installation process is needed. Download PTMiner.zip and extract it to an appropriate directory. Then, double click *PTMiner.exe* to start PTMiner.

## PROTOCOL

### About the construction of this protocol

Since PTMiner performs differently on open-search results and closed-search results, we will describe the two procedures separately.

### Procedures for open search

#### Step 1: Start the PTMiner software

Double click *PTMiner.exe* and start the PTMiner software.

**[Note]** A “Windows protected your PC” dialog may pop up when opening PTMiner for the first time because Microsoft Defender SmartScreen cannot recognize the publisher of the program. Just click “More info” and choose “Run anyway” to open the main interface of PTMiner.

#### Step 2: Set parameters in the “Search Parameters” tab

**Figure 2:**
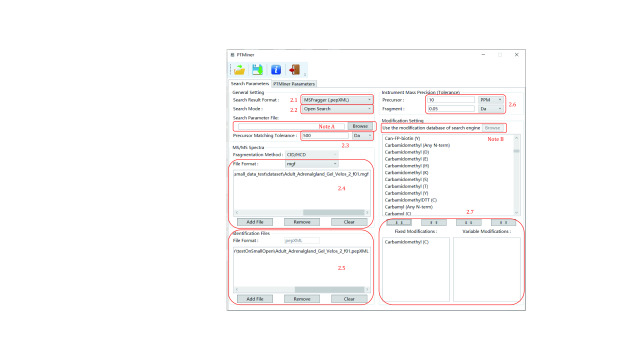
Setting the search parameters (open search)

Set the same parameters as used by the search engine in the “Search Parameters” tab (Fig. 2).

Step 2.1: Select “Search Result Format” according to your search engine. PTMiner supports the result format of pFind 3, pFind 2.8, MSFragger, Comet, Sequest and PTMiner’s own format.

**[Note]** The search results of MS-GF+ or MaxQuant can be converted to PTMiner format using the tool *PSMConvert.exe* provided in the PTMiner directory. For MS-GF+ in particular, before converting to PTMiner format, the identification result file (.mzid) should be converted to a tab-separated file (.tsv) with tools described in the MS-GF+ usage information. When converting a .tsv file to PTMiner format using *PSMConvert.exe*, the specified modification file should follow the format of *MSGFPlus_Mods1.txt* provided in the MS-GF+ package.

Step 2.2: Select “Open Search”.

**[Note]** Open search is also called mass-tolerance search, in which a large precursor tolerance widow, *e*.*g*., 500 Da, is used. For pFind 3 in particular, Open Search contains two modes, *i*.*e*. “Open search (Open-pFind)” and “Open Search (large tolerance)”. The former was designed for Open-pFind (Chi* et al.* 2018), in which the “open search” checkbox of pFind 3 is checked and a small, normal tolerance is used. PTMiner performs similarly to the closed search mode (refer to section 3.3) when “Open search (Open-pFind)” is selected. The latter was designed for mass-tolerant search of pFind 3, which means that the “open search” checkbox of pFind 3 is not checked and a large precursor mass tolerance, *e*.*g*., 500 Da, is used. This is the open search mentioned in this section.

**[Note]** PTMiner can be seamlessly connected to the downstream analysis for results of pFind 3 and MSFragger since most search parameters can be imported by loading the configuration file of the search engine. Click “Browse” beside the “Search Parameter File” and choose the *\param\pFind.cfg* for pFind 3 or .*params* for MSFragger to automatically set the search parameters (“Note A” in Fig. 2). For pFind 3, loading the modification database used by pFind3 is also supported: click “Browse” to select the *modification.ini* file in the pFind 3 directory (“Note B” in Fig. 2).

Step 2.3: Specify the “Precursor Matching Tolerance”. The value of this parameter must be consistent with that used by the search engine.

Step 2.4: Select the corresponding MS/MS spectra file. Click the “Add File” button to add MS/MS Spectra. If incorrect files are selected, click the “Remove” button to delete the file(s) selected in the list, or click the “Clear” button to delete all files. As an example, a .mgf file (\demo\small_data_test\dataset\Adult_Adrenalgland_Gel_Velos_2_f01.mgf) is loaded.

**[Note]** PTMiner only supports centroid MS/MS spectra in .mgf format. If the search engine searched spectra that were already in .mgf format, then the same .mgf files should be used by PTMiner. Otherwise, the spectra must be converted to .mgf format properly. For example, using the MSConvert tool (Chambers* et al.* 2012) with default configuration can meet the requirements of format conversion. For MaxQuant, there must be the “SCAN=” value in the “TITLE” attribute in the .mgf files. For MSFragger version 3.4 or higher, if raw files are searched, .mgf files with the ‘_*uncalibrated*’ suffix in their file names will be generated by default in the *raw* directory. Either these .mgf files with the ‘_*uncalibrated*’ suffix removed or .mgf files converted from raw files using MSConvert can be specified as the spectra files for PTMiner. For pFind 3, if raw files are searched, .mgf files will be generated by default (saved in the same directory as raw files), and these .mgf files should be used by PTMiner. However, importantly, only one single integrated .mgf file is supported for pFind3 and thus multiple .mgf files should be combined into one using the *FilesMerger.exe* tool provided in the PTMiner directory.

Step 2.5: Select the search result of the search engine in the “Identification Files” region. PTMiner supports .spectra format for pFind 3, .txt format for pFind 2.8, .pepXML format for MSFragger, .pep.xml format for Comet, .txt format for SEQUEST, and PTMiner .txt format. For example, a .pepXML file (demo\small_data_test\open\MSFragger\Adult_Adrenalgland_Gel_Velos_2_f01.pepXML) is loaded here.

Step 2.6: Specify the precursor and fragment tolerances according to the instrument mass precision.

**[Note]** The fragment tolerance can be the same as that used by the search engine, but the precursor tolerance may be different. For open search, the precursor tolerance here is not the large tolerance used by the search engine but rather the normal tolerance, *e*.*g*., 20 ppm.

Step 2.7: Specify the same fixed and variable modifications as used by the search engine.

**[Note]** For pFind3, we recommend adding all modifications including fixed and variable modifications into the “variable modification” list of PTMiner.

#### Step 3: Set FDR filtering parameters

**Figure 3:**
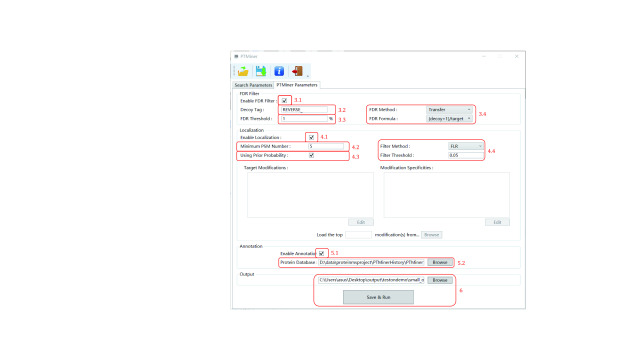
Setting the PTMiner parameters (open search)

Set the FDR filtering parameters as shown in Fig. 3.

Step 3.1: Specify whether to do FDR estimation and PSM filtering.

**[Note]** By default, “Enable FDR Filter” is checked. Uncheck it if you want to skip the FDR filtering step, which means that all PSMs from the search result will undergo the localization step.

Step 3.2: Specify the “Decoy Tag” used by the search engine to indicate decoy sequences in protein names.

Step 3.3: Specify the “FDR Threshold”.

Step 3.4: Select the “FDR Method” and “Target Decoy Method”.

**[Note]** We recommend the transfer FDR estimation method, which performs robustly when the modification group is small, and the (decoy + 1)/target estimation formula, which strictly controls the FDR (He* et al.* 2015).

#### Step 4: Set localization parameters

Set the localization parameters as shown in Fig. 3, also.

Step 4.1: Specify whether to re-localize mass shifts.

**[Note]** By default, “Enable Localization” is checked. Uncheck it if you want to skip this step, in which case the search engine-determined locations (if available) will be used.

Step 4.2: Specify the “Min Modification Number” to filter out infrequent mass shifts.

**[Note]** The default value of 5 means that PTMiner will ignore mass shifts that occur in less than 5 PSMs

Step 4.3: Specify whether to use the prior probability during localization.

**[Note]** By default, “Using Prior Probability” is checked for open search, indicating that the prior probabilities will be iteratively updated and PTMiner makes use of both the spectral peak matching information and the site specificity preference learned from the data to localize the mass shifts. Unchecking the checkbox implies that PTMiner exclusively relies on the peak matching information for localization.

Step 4.4: Specify the “Filter Method” and “Filter Threshold”. The “Filter Method” contains two options, namely “Probability” and “FLR”. The former is the posterior probability of the localized site (which is shown in the result file), and the latter is the false localization rate. Localization results with probability or FLR less significant than the threshold will be filtered out.

#### Step 5: Set annotation parameters

Likewise, set the localization parameters as shown in Fig.3.

Step 5.1: Specify whether to annotate the mass shifts.

**[Note]** By default, “Enable Annotation” is checked. Uncheck it if you do not want to perform annotation.

Step 5.2: Click “Browse” and select the protein sequence database file (in .fasta format) that is used during the database search. For instance, a .fasta file (\demo\database\uniprot-sp-human.fasta) is loaded.

**[Note]**: In addition to matching the mass shifts to known modifications, PTMiner also tries to interpret the mass shifts as possible amino acid variations. This is why the sequence database is needed.

#### Step 6: Set output directory and run

After setting the output directory, a parameter file will be generated and saved before running (Fig. 3).

#### Step 7: View the running results

Open the output directory to view the running results.

**[Note]** If the “Enable FDR Filter” is checked, *filtered_result.txt* and *filtered_summary.txt* will appear in the directory. *loc_result.txt* and *loc_summary.txt* will appear in the directory if “Enable Localization” is checked. Furthermore, *anno_result.txt* and *anno_summary.txt* will appear when “Enable Annotation” is checked. These files are tab-delimited text files and can be properly opened and viewed with Microsoft Excel.

**Figure 4:**
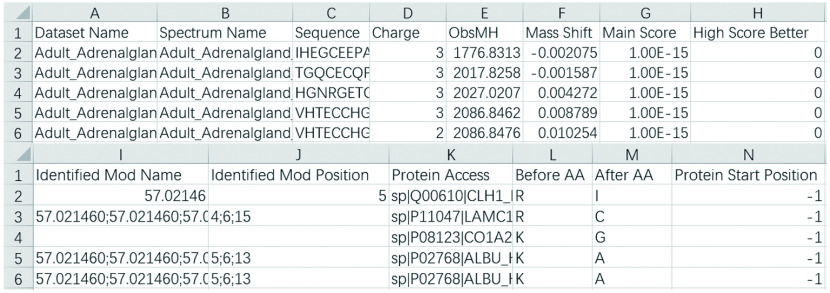
An example of *filtered_result.txt* displayed by Excel. Since one line of the original result file is too wide to display, it is cut into two lines to show here

Step 7.1: Open the *filtered_result.txt*. An example is shown in Fig. 4. The meaning of each column is given in Table 1.

**Table 1:** The meanings of columns in *filtered_result.txt*

Column	Meaming
Dataset name	Spectrum file name without path, *e*.*g*., “test.mgf”
Spectrum name	Spectrum name or scan number
Sequence	Identified peptide sequence
Charge	Precursor charge
ObsMH	The experimentally observed peptide MH+
Mass shift	Mass difference between observed and identified peptides
Main score	*Final score* for pFind 3, *E-value* for pFind 2.8, *expect search score* for MSFragger and Comet, *XCorr* for SEQUEST, *SpecEValue* for MS-GF+ , *Score* for MaxQuant
High score better	Whether higher main score is better. 1 for yes and 0 for no
Identified Mod name	Identified modification names including both fixed and variable modifications. If the PSM contains more than one modifications, they are separated by “;”, *e*.*g*., “Carbamidomethyl;Carbamidomethyl”. If the PSM contains no modifications, it is empty
Identified Mod position	Identified modification positions corresponding to the identified modifications. If the PSM contains more than one modifications, they are separated by “;”, *e*.*g*., “6;12”. If the PSM contains no modifications, it is empty. “0” represents the N-terminal of the peptide and “length of peptied + 1” represents the C-terminal of the peptide, 1 to “length of peptide” indicate the corresponding position of the peptide sequence
Protein access	The access of protein from which peptide comes. If the PSM contains more than one proteins, they are separated by “;”, *e*.*g*., “sp|P05141|ADT2_HUMAN;sp|P12236|ADT3_HUMAN”
Before AA	The adjacent amino acid before peptide in protein. If the PSM contains more than one proteins, they are separated by “;”, *e*.*g*., “K;K”. Otherwise it is set to “-”
After AA	The adjacent amino acid after peptide in protein. If the PSM contains more than one proteins, they are separated by “;”, *e*.*g*., “R;H”. Otherwise it is set to “-”
Protein start position	The starting position of the peptide in the protein sequence. It is only available when the search engine is pFind 3 or pFind 2.8. Otherwise, it is set to –1

**Figure 5:**
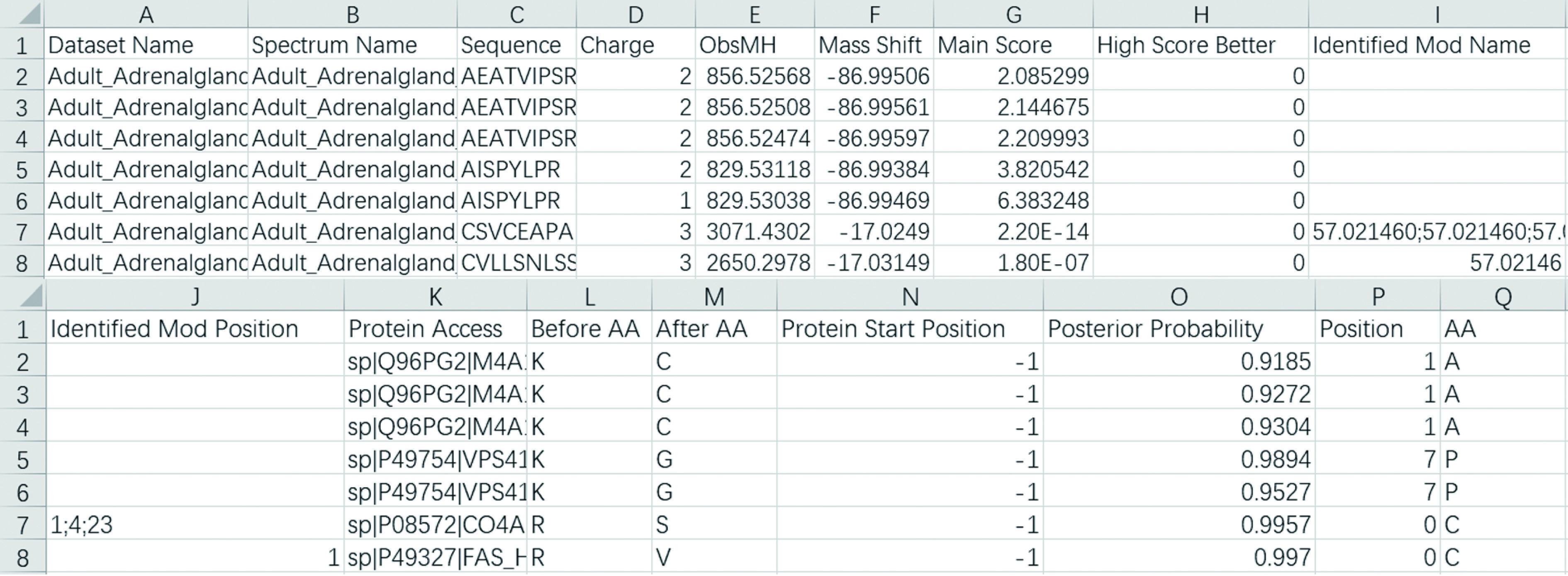
An example *loc_result.txt* displayed by Excel. Since one line of the original result file is too wide to display, it is cut into two lines to show here

Step 7.2: Open the *loc_result.txt*. An example is shown in Fig. 5. The meanings of the columns that appeared in *filtered_result.txt* are given in Table 1, and the meanings of additional columns are given in Table 2.

**Table 2:** The meanings of additional columns in *loc_result.txt*

Column	Meaning
Posterior probability	The maximum posterior probability of the position localized in the peptide sequence
Position	The position with the maximum posterior probability
AA	The amino acid with the maximum posterior probability

**Figure 6:**
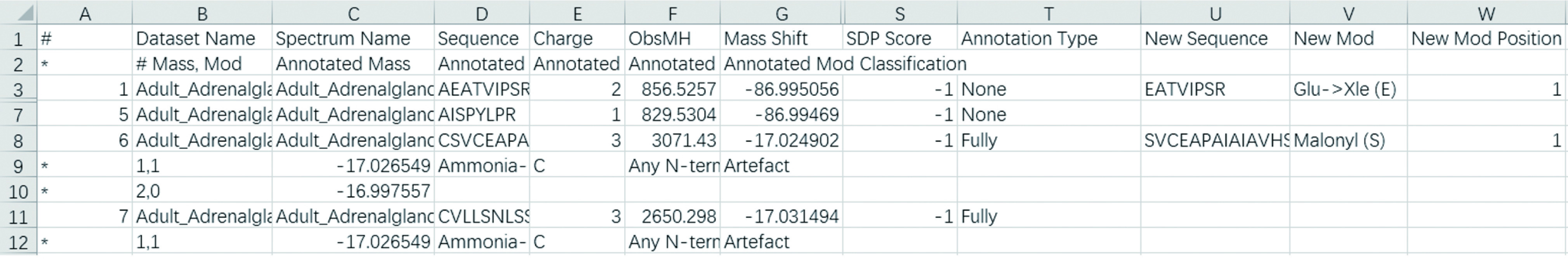
An example* anno_result.txt* displayed by Excel. Since one line of the original result file is too wide to display, the middle part of the lines are omitted here

Step 7.3: Open the *anno_result.txt*. An example is shown in Fig. 6. *Anno_result.txt* contains two headers. The first header is for basic information on the localization result (the first line in Fig. 6). Lines starting with a number corresponding to it. The second header is for the annotation result (the second line in Fig. 6) which corresponds to lines starting with asterisks. The meanings of columns in the first header that appeared in* loc_result.txt* are given in Table 1 and Table 2, and the additional columns are given in Table 3 and Table 4.

**Table 3:** The meanings of additional columns in the first header in *anno_result.txt*

Column	Meaning
SDP score	Similarity score between spectra of modified and corresponding unmodified peptides (with the same sequence and non-target modifications)
Annotation type	Including three types, *i*.*e*. “Fully”, “Partially” and “None”. “Fully” indicates that both mass shift and site specificity are matched to existing modification(s) in Unimod. “Partially” indicates that only the mass is matched. “None” represents that the mass shift cannot be found in Unimod
New sequence	Updated new sequence after deleting or adding some amino acids on peptide termini. If a new peptide sequence (with or without modification) explains the mass shift better, this column will show the sequence. Otherwise, it is empty
New Mod	The possible explanation of the new mass shift given the new sequence above
New Mod position	The position of the new modification above

**Table 4:** The meanings of columns in the second header in *anno_result.txt*

Column	Meaning
*	This sign indicates that this row is annotation result
# Mass, Mod	The order of the annotated mass (Mass) and the order of the annotated modification (Mod)
Annotated mass	Annotated mass
Annotated Mod	Annotated modification
Annotated Mod site	The same as the “Site” in the Unimod database
Annotated Mod term Spec	The same as the “Position” in the Unimod database
Annotated Mod classification	The same as the “Classification” in the Unimod database

**Figure 7:**
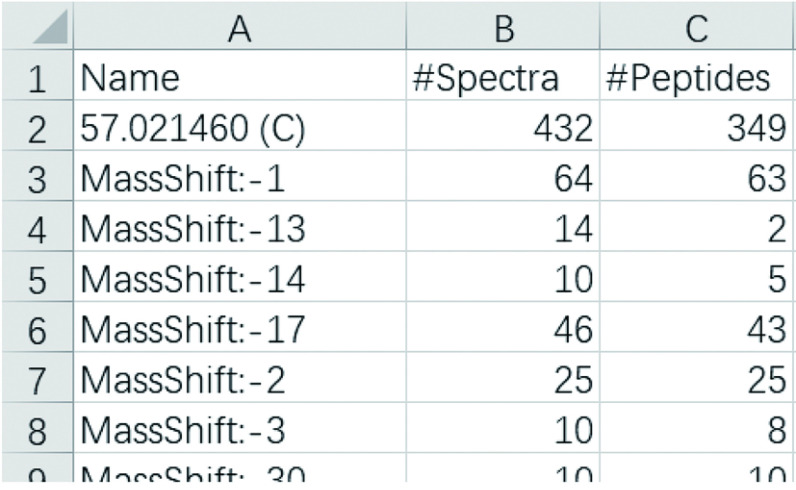
An example* filtered_summary.txt* displayed by Excel

Step 7.4: Open the *filtered_summary.txt*. An example is shown in Fig. 7. The meanings of columns are given in Table 5. Open the *loc_summary.txt* and *anno_summary.txt* to view data in them. The columns in *loc_summary.txt* and *anno_summary.txt* have the same meaning as those in *filtered_summary.txt*. The former summarizes the modifications in *loc_result.txt*, while the latter summarizes the modifications in *anno_result.txt*.

**Table 5:** The meanings of columns in *filtered_summary.txt*

Column	Meaning
Name	The names of all modifications that appear in the filtered_result.txt. For open search mode, a mass shift will also be treated as a modification for statistics, and will be displayed in the form of “MassShift:X”, where X is an integer obtained by rounding the mass shift
#Spectra	The number of spectra with the above modification
#Peptides	The number of peptides with the above modification. Peptides are considered as the same when their peptide sequences and modification(s) (including site(s)) are the same
#Sites	The number of sites with the above modification. Sites are considered as the same, if their protein sets (protein accession and modification position on the protein sequence) given in the identification result are the same. This column will only appear when the search engine is pFind 3 or pFind 2.8. Since no location information can be acquired in the FDR filtering step, “MassShift:X” does not have a site-level count in the results of open search

### Procedures for closed search

#### Step 1: Start the PTMiner software

Double click *PTMiner.exe* and start the PTMiner software.

#### Step 2: Set parameters in the “Search Parameters” tab

**Figure 8:**
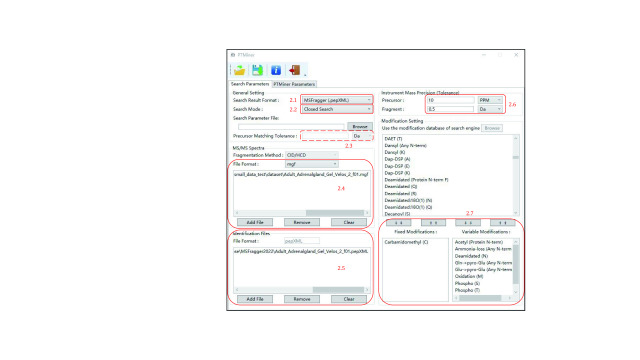
Setting the search parameters (closed search)

Set the same parameters as used by the search engine in the “Search Parameters” tab (Fig. 8). Steps 2.1–2.7 are almost the same as described in Step 2 of the open search mode. The only difference is that the third substep, *i*.*e*., setting the “Precursor Matching Tolerance”, is not required. As an example, the same .mgf file as Step 2.4 in Section 3.2 and a .pepXML file (\demo\small_data_test\close\MSFragger\ Adult_Adrenalgland_Gel_Velos_2_f01.pepXML) are loaded.

**[Note]** Automatically setting parameters by loading the configuration file of the search engine also works for the closed search mode. Click the “Browse” button beside the “Search Parameter File” label in the “Search Parameters” tab to load the configuration file. The instructions are the same as those for the open search mode (“Note A” in Fig. 2).

#### Step 3: Set FDR filtering parameters

**Figure 9:**
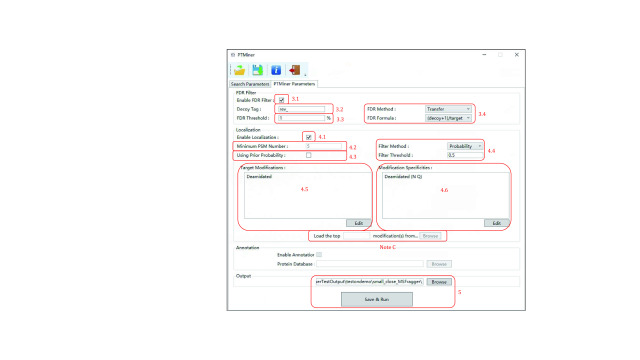
Setting the PTMiner parameters (closed search)

Set the FDR filtering parameters (Fig. 9). Steps 3.1–3.4 are the same as described in Step 3 of the open search mode.

#### Step 4: Set localization parameters

Set the localization parameters as shown in Fig. 9. Steps 4.1–4.4 are the same as described in Step 4 of the open search mode. We recommend unchecking “Using Prior Probability” for closed searches.

**[Note]**: For pFind 3, click “Browse” (“Note C” in Fig. 9) to select the *\result\pFind.summary* for automatically loading the modifications and specificities in 4.5 and 4.6, specify the “top N” blank indicate loading the N most abundant modifications.

**Figure 10:**
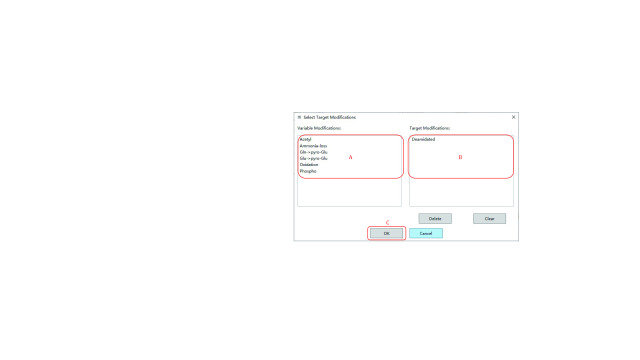
Selecting the target modifications

Step 4.5: Click “Edit” in the “Target modifications” region in Fig. 9 to specify the target modifications, and a “Select Target Modification” dialog box shown in Fig. 10 will pop up.

(A) Specify the target modifications of interest in the “Variable Modification” list (candidate modification list). Once a modification is selected, it will move from the “Variable Modification” list to the “Target Modifications” list (selected modification list).

(B) Check if the “Target Modifications” list is exactly composed of all the modifications of interest. If the “Target Modifications” list contains unnecessary modifications, click “Delete” to remove the modifications selected in the list or click “Clear” to remove all modifications in it.

(C) Click “OK” to complete the specifying of the target modifications.

**Figure 11:**
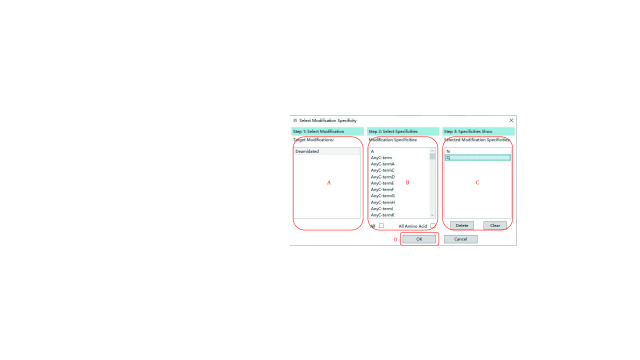
Selecting the site specificities for each modification

Step 4.6: Click “Edit” in the “Modification Specificities” region in Fig. 9 to specify at least one possible site specificity for each target modification, and PTMiner will only localize modification on sites that meet the site specificity condition. After clicking the “Edit” button, a “Select Modification Specificity” dialog box, as shown in Fig. 11, will pop up.

(A) Select a modification in the “Target Modifications” list (left) to show the candidate and selected specificities list of this modification. The list of candidate specificities is shown in the “Modification Specificities” list (center), and the list of selected specificities is shown in the “Selected Modification Specificities” list (right).

(B) Click on all possible site specificities in the “Modification Specificities” list to move them to the “Selected Modification Specificities” list, or click “All” at the bottom to select all specificities, or click “All Amino Acid” at the bottom to select all amino acids as selected specificities.

(C) Check if the “Selected Modification Specificities” list is exactly composed of all the specificities of interest. If the list contains unnecessary specificities, click “Delete” to remove specificities selected in “Selected Modification Specificities” or click “Clear” to remove all specificities in it.

(D) Click “OK” to complete the specifying of the site specificities for all target modifications.

Annotation is not needed for closed search and is disabled.

#### Step 5: Set output directory and run

After setting the output directory, a parameter file will be generated and saved before running (Fig. 9).

#### Step 6: View the running results

**Figure 12:**
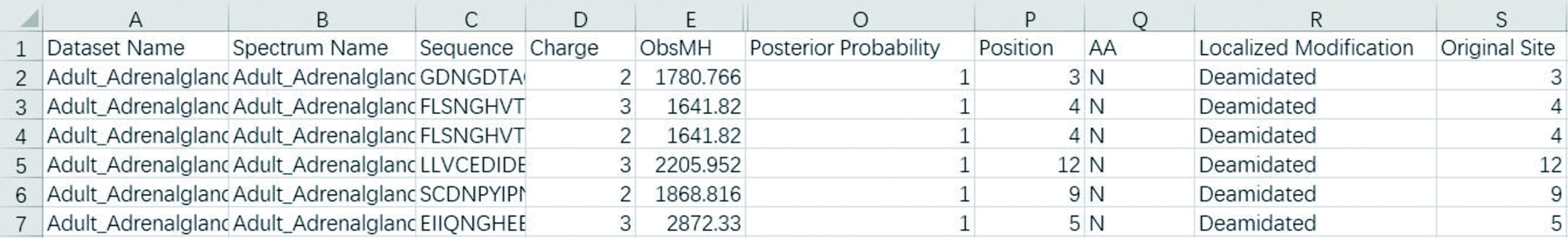
An example* loc_result.txt* displayed by Excel. Since one line of the original result file is too wide to display, the middle part of the lines are omitted here

Open the output directory to view the running results. The format of the files is almost the same as described in Step 7 of the open search. The only difference is that the two columns appended to the right of the *loc_result.txt* file in closed search mode (Fig. 12). These two columns are “Localized Modification” and “Original Site” respectively. “Localized Modification” represents the name of the target modification provided by the search engine, and “Original Site” indicates the original site of modification localized by the search engine.

## Conflict of interest

Zhiyuan Cheng, Ge Song and Yan Fu declare that they have no conflict of interest.
